# Roots of responses: A qualitative study on intergenerational transmission of illness approaches

**DOI:** 10.1016/j.ijchp.2026.100692

**Published:** 2026-05-30

**Authors:** Elske Hogendoorn, Sterre van der Ziel, Aranka V. Ballering, Donald G. van Tol, Judith G.M. Rosmalen

**Affiliations:** a University Medical Center Groningen, Department of Psychiatry, Netherlands; b University of Groningen, Department of Sociology, Netherlands; c University Medical Center Groningen, Department of Primary and Long-term care, Netherlands; d University Medical Center Groningen, Department of Internal Medicine, Netherlands

**Keywords:** Intergenerational transmission, Illness, Physical symptoms, Parent-child interactions, Qualitative, Interviews, Symptom responses

## Abstract

**Background:**

Substantial evidence supports the intergenerational transmission of proneness to physical symptoms. In line with social learning theory, parent-child interactions significantly influence children’s symptom experiences and coping strategies. However, it remains unclear how these childhood interactions translate into illness-related parenting later on. This study aims to elucidate how childhood illness-related interactions with parents shape individuals’ approaches to their own children’s illness.

**Methods:**

We employed a qualitative design, integrating epidemiological data from the Lifelines Cohort to purposively sample 32 adult participants based on their own and their parents’ symptom burden. Participants (53% male, aged 28-74) were interviewed using a semi-structured guide exploring experiences with illness in childhood and parenting. Transcripts were coded using a mixed deductive-inductive approach and analyzed thematically focusing on processes of modeling and reinforcement. We included a deviant case analysis.

**Results:**

Three parental symptom response types emerged: affective, practical, and minimizing. These were mirrored or contrasted in participants’ own caregiving. Affective and practical responses were generally evaluated positively or neutrally, while minimizing responses were often viewed negatively and linked to deliberate divergence. Participants described value-driven examples they aimed to model, categorized as transparent and vulnerable, stable and calm, or tough and perseverant. Values did not always align with actual symptom responses, revealing discrepancies between intended modeling and behavioral reinforcement.

**Conclusions:**

Intergenerational transmission of illness approaches varies greatly between families and is influenced by personal experiences, values and context. These findings highlight the complexity of intergenerational transmission and may inform family-based interventions to support adaptive coping in children.

## Introduction

Physical symptoms, such as headache, fatigue or nausea, are symptoms that can arise in the absence or presence of an established medical condition. The occurrence of physical symptoms is multifactorial, involving a complex interplay of biological, psychological and social factors (Kitselaar et al., 2023). Symptoms can be transient and short-lived or persist for longer periods of time. Experiencing persistent physical symptoms has been associated with work absenteeism, heavier reliance on the medical system and a decreased quality of life (Chaabouni et al., 2024; Joustra et al., 2015; Tomenson et al., 2013). A substantial variability in the occurrence of physical symptoms is present among individuals, but also in the associated subjective distress and the coping strategies adopted in response to those symptoms (Ballering et al., 2022; Eliasen et al., 2016).

A known risk factor for suffering from persistent physical symptoms is having a parent who suffers from a high physical symptom burden, indicating an intergenerational transmission of physical symptom proneness (Dario et al., 2019; Hestbaek et al., 2004; Higgins et al., 2019). Intergenerational transmission may be symptom-specific. As such, it has been found that mothers with abdominal pain are more likely to have children who also report abdominal pain (Ramchandani et al., 2006; Saunders et al., 2007). However, intergenerational transmission patterns that are not symptom-specific have also been shown, for instance, mothers with abdominal pain are more likely to have children with headache, fatigue and muscle soreness (Levy et al., 2004; van Tilburg et al., 2015). This suggests that parents may transfer a general susceptibility to experiencing physical symptoms to their children.

Although intergenerational transmission of physical symptom proneness could occur via genetic pathways (Kato et al., 2010; Vehof et al., 2014), social learning processes may also play a role. Through everyday parent-child interactions, children learn to perceive, interpret and manage symptoms (van Tilburg et al., 2015). Two key social learning mechanisms within these interactions are modeling and reinforcement. Modeling refers to examples parents set with their own behaviors, e.g., staying home from work when having a headache, which children observe and imitate. Reinforcement involves parental responding to behaviors of the child, e.g., taking a child with a headache to the doctor, shaping the child’s future behavior through consequences (Bandura, 1977). Experimental research shows associations between parental symptom modeling and responses, and children’s symptom-related distress, for example, in a study where mothers and children both participated in a cold pressor task. In the group of mothers who were instructed to exaggerate their pain expression, children also expressed more pain when they performed the task thereafter, while this was not observed in the control group (Goodman & McGrath, 2003). This shows the impact of parental pain modeling on children’s pain experiences. Naturalistic observation studies have reported associations between parental responses and children’s pain during vaccinations (Lisi et al., 2013; Sobol-Kwapińska et al., 2020). Similarly, protective parental responses to non-acute symptoms (e.g., keeping a child home for a stomachache) are associated with increased symptom reporting (Janssens et al., 2009; Simons et al., 2008). Longitudinal general population cohort studies have shown long-term influences of parental health-related factors, such as medication use and healthcare utilization, on children’s physical symptoms years later (Burke et al., 2025; Hogendoorn et al., 2024). This body of literature illustrates the important role of parent-child interactions in the child’s symptom experiences.

The influence of parent-child interactions on the child’s symptom experiences may occur through the shaping of the child’s illness approach. We define an illness approach as a person’s organized set of ideas, feelings and management strategies regarding health, illness and disease. It encompasses attitudes, behaviors, and decision-making processes regarding health and illness. This includes how people interpret (the severity of) symptoms, when and why they seek medical advice, how they attempt to maintain good health, and how they communicate about health- and illness-related matters within their family. The concept ‘illness approach’ relates to, but is broader than, established constructs such as illness perceptions, health beliefs, or coping styles (Bakken et al., 2023; Bunzli et al., 2021; Green et al., 2020). Whereas these frameworks typically address specific cognitive or behavioral components, an illness approach captures an overarching orientation towards health and illness. It is also distinct from the concept of illness mindset, which focuses on core assumptions about chronic illness within defined patient groups (Yielder et al., 2026). In contrast, illness approaches provide a comprehensive concept applicable across individuals of the general population with varying illness experiences. Through social learning mechanisms, such as verbal communication, modeling, and reinforcement of symptom-related behavior, parents’ own illness approaches may shape those of their offspring. This could in turn influence the offspring’s future health and illness-related interactions with their own children when they become parents.

So far, most qualitative studies about interactions related to physical symptoms in the family context focused on chronic illness. Studies in the general population are scarce. One interview study involving mother-adolescent dyads from the general population explored how information about pain and pain management is transmitted from parent to child (Hatchette et al., 2006). Both mothers and adolescents highlighted verbal communication and modeling as pathways that could contribute to shared attitudes towards pain approaches. Most mothers in this sample were aware of their function as role models and articulated making conscious decisions in whether or not to express their own pain and in showing coping strategies in the presence of their child. Similarly, in a focus group study about communication of everyday pain in young children, parents described the influence of their own modeled pain expression on their child’s pain communication. They deliberately chose whether to openly discuss being ill with their children or to hide it (Liossi et al., 2012).

Yet, it remains largely unknown how such childhood interactions translate into a person’s illness approach in adulthood and how this may be transferred to the next generation. It is unclear if and why individuals adopt similar or different illness approaches to those of their own parents, and how individuals evaluate these approaches. Gaining insight into how illness and health are experienced and approached across generations could provide a deeper understanding of the pathways of intergenerational transmission of proneness to physical symptoms. Studies in non-clinical samples exploring a broad spectrum of everyday symptoms could enhance the applicability of findings beyond clinical populations, possibly yielding insights into patterns related to symptom coping and healthcare utilization that are relevant to a wider population.

The aim of the current study was to elucidate how health- and illness-related interactions with parents in childhood shape how individuals approach illness in their own children when they have become parents. To this end, we conducted qualitative interviews in a sample of 32 adult participants drawn from a large general population cohort.

## Methods

This study is part of a larger research project aimed at exploring various aspects of illness perception, interpretation, management, and communication within the family context. The overarching research project is designed to address multiple research questions. Given the project's aim to understand the lived experiences of families, with an emphasis on the ideas and reasons behind these encounters, a qualitative study design is considered most appropriate (Hennink, Monique et al., 2020). The current study focuses on a specific selection of these data. The overarching study aims, design, data collection procedures and successive versions of the interview guide have been preregistered at the Open Science Framework (https://osf.io/a4gn2).

This study was conducted in line with the Consolidated criteria for Reporting Qualitative Research (COREQ) checklist (see Appendix) (Tong et al., 2007).

### Participants

Participants were purposively sampled from the Lifelines Cohort Study. Lifelines is a multidisciplinary prospective population-based cohort study examining in a unique three-generation design the health and health-related behaviors of 167,729 persons living in the North of The Netherlands. It employs a broad range of investigative procedures in assessing the biomedical, sociodemographic, behavioral, physical, and psychological factors which contribute to the health and disease of the general population (Scholtens et al., 2015). The Lifelines Cohort Study is performed according to the principles of the Declaration of Helsinki and in accordance with the research code of the University Medical Center Groningen (UMCG). This Lifelines add-on study was approved by the Central ethics Review Board of the UMCG, The Netherlands (202200104).

For the current study, we included the second generation of three-generation Lifelines families. Thus, participants were adult Lifelines participants, whose parents were also enrolled in Lifelines and who had one or more children. Participant selection was further based on physical symptom burden over time, as assessed in a previous Lifelines study (Ballering et al., 2022). These trajectories of physical symptom burden enabled us to specifically select and interview families with various symptom burdens over time and across generations. Participants were classified as having a high or a low symptom burden. Subsequently, we defined the following four groups: 1. The participant and at least one parent had a high symptom burden; 2. The participant had a low symptom burden and at least one parent had a high symptom burden; 3. The participant and both parents had a low symptom burden; 4. The participant had a high symptom burden and both parents had a low symptom burden. Since the groups of participants with a high symptom burden (1 and 4) were small, we also invited participants who had a high symptom burden but whose parents' burden was unknown. No selection criterion was defined based on the age or symptom patterns of the child(ren) of the participants (third generation). Furthermore, we purposively sampled for sufficient variation in age, sex, socioeconomic status and living environment (rural/urban) of the participants. More detailed information on the sampling method and descriptive characteristics of the participants, such as occupations, have been reported elsewhere (Ballering et al., 2026).

### Data collection

Data were collected by conducting face-to-face, in-depth interviews. A semi-structured interview guide was developed by the multidisciplinary research group based on literature and group discussion. The interview guide was rooted in the main overarching research aims and contains questions about perception, interpretation, management and communication of symptoms, as experienced in the family context. Questions addressed both past experiences in the participant’s childhood and upbringing by their parents, as well as present experiences and upbringing of the participant’s own child(ren). The full interview guide can be accessed via the preregistration (https://osf.io/a4gn2).

The research team consisted of six researchers with different backgrounds, being: medical biology and psychology (JR, female); sociology (DT, male); medicine (SZ, female); pedagogy (EH, female); biomedical sciences (AB, female); and developmental psychology (SB, female). Some had no prior experience with qualitative research, while others had over 15 years of experience. Interviews were conducted by five members of the team (DT, SZ, EH, AB and SB). Different interviewers have different styles of interviewing, connotations with the narratives, triggers, and effects on the participants, resulting in variation of the data obtained through the interviews. To ensure a base level of consistency between the interviews, we used a uniform interview guideline, participated in an actor-based interview training together and discussed all interviews within the team.

Each interviewer conducted a pilot interview to test the usability of the interview guide, as well as to further refine interviewing skills. Pilot participants were non-Lifelines participants that were recruited through personal networks using convenience sampling. Following the pilot interviews, amendments to the interview guide primarily included minor rephrasing of questions and adding additional questions and probes. Data obtained from the pilot interviews were not included in the study.

After the pilot interviews, eligible Lifelines participants were approached via a letter containing information about the study’s purpose, accompanied by an informed consent form. If participants were willing to be interviewed, they could send the completed informed consent forms to Lifelines. The researchers then contacted participants by telephone to set an appointment for the interview. Each participant was interviewed during one session of approximately one hour, taking place at a location of the participant's choice. This could be at the participant’s house, at their workplace or at the interviewer’s office. Three interviews were conducted online via Microsoft Teams. All interviews were audio-recorded.

We kept a log file including the research steps taken from the start of a research project to the development and reporting of findings. The preregistration served as an open audit trail in which changes to the interview guide were documented.

### Analysis

The interviews were transcribed verbatim using Amberscript transcription software and anonymized by the interviewer. Short fieldnotes that were made after each interview were added to the transcript to provide context. Amberscript transcriptions were checked and corrected by the researcher if necessary, aiding in their familiarization with the interviews. The transcripts were coded using Atlas.ti version 24. We conducted a member check by providing all interviewed participants with their verbatim transcripts and allowing them to return any corrections or additions.

We developed a coding scheme consisting of deductive codes based on the literature and expertise of the team members. This scheme was subsequently expanded with inductive codes during an iterative process and by applying the method of constant comparison while data collection was still ongoing (Hennink et al., 2020). Coding of the transcripts was performed by four members of the team (EH, SZ, AB and DT). Each transcript was independently coded by two researchers, with varying researcher pairs to establish a shared interpretation framework in the team, applied by all team members in a more or less similar manner. After independent coding, codes of the different coders were compared and consensus was reached during discussion. Discussion points were noted in a log file. In team meetings during the first coding round, the entire project group reflected on the interviews, interpretations of the text and connections between the data. After coding 15 to 20 interviews, the first signs of saturation became apparent as no new codes emerged (Hennink et al., 2017; Rahimi & Khatooni, 2024). However, we continued to recruit participants to ensure that all four groups of family symptom burden were represented in the sample, aiming to capture sufficient variation in illness approaches.

During the first round of coding sessions and team discussions within the larger research group, several broad themes emerged from the data. EH and SZ focused on the theme ‘intergenerational transmission’ in a second round of coding, the central topic of this sub-study, while other themes were further developed in analyses of the related sub-studies. Two key aspects of social learning, reinforcement and modeling, were explored. Certain codes of interest (e.g., ‘managing child symptoms by parent’, related to reinforcement, and ‘setting an example’, related to modeling) were refined in order to analyze these data on a more detailed level.

Subsequently, the thematic analysis method One Sheet Of Paper (OSOP) was employed (Ziebland & McPherson, 2006). We used this method to visually organize and integrate categories and identify themes with regard to health- and illness-related interactions with parents and intergenerational transmission of illness approaches. This technique involves reviewing all quotes that were assigned a specific code, and noting them on a single sheet of paper, organized based on similarities and differences. This facilitates identifying meaningful patterns and themes (Ziebland & McPherson, 2006). Different OSOP visualizations were made for ‘parental responses to participants’ symptoms’, ‘participants’ responses to their own children’s symptoms’ and ‘examples by participants’. Subsequently, through an iterative process, themes were identified and patterns of intergenerational transmission were explored through team discussions and visualizations mapping pathways of illness approaches across the generations. A log file was kept to document the analyzing process, including decisions that were made and the reasoning behind them.

Lastly, we performed a deviant case analysis in which we explored elements in the data that seemingly contradicted the emerged patterns of intergenerational transmission.

We used quotes to illustrate the findings. Quotes were translated verbatim to stay as close to the participants’ original wording as possible. The untranslated Dutch quotes can be found at the OSF (https://osf.io/a4gn2).

## Results

Thirty-two adults were interviewed. General sociodemographic characteristics of the sample are shown in Table 1.

**Table 1:** Overview of the sociodemographic characteristics of the participants.

			Participants (N=32)
**Sex, N (%)**	Male		17 (53.1)
	Female		15 (46.9)
**Age in years, M (SD)**			47.7 (10.7)
**Educational level, N (%)**^a^	Low to medium		19 (59.4)
	High		13 (40.6)
**Geographical location, N (%)**^b^	Extremely urbanized		3 (9.4)
	Strongly urbanized		5 (15.6)
	Somewhat urbanized		4 (12.5)
	Strongly rural		5 (15.6)
	Extremely rural		15 (46.9)
**Familial symptom burden, N (%)**	Participant	Parent	
	High	High	2 (6.3)
	Low	High	14 (43.7)
	Low	Low	4 (12.5)
	High	Low	9 (28.1)
	High	Unknown	3 (9.4)

^a^This table was based on a table published elsewhere (Ballering et al., 2026)

^b^Categorization of educational level in Lifelines is defined elsewhere (van Zon et al., 2017)

^c^Categorization of urbanization is defined by Statistics Netherlands (Statistics Netherlands, 2023)

### General findings

All 32 participants shared reflections about their childhood experiences with health and illness in the family, as well as their approaches to the illness-related upbringing of their own children. These narratives emerged both in response to direct questions and through spontaneous sharing. We observed considerable variation among participants in terms of symptom burden, personal and familial illness histories, the quality of family relationships, and how central the topics of health and illness were to their lives. In general, good rapport was established and participants spoke candidly about their experiences with symptoms and illness in their families.

When discussing their partners, participants attributed different roles to them in relation to approaching their child’s illness. Some partners acted as extensions of the participant, sharing similar ideas and strategies. Other partner adopted a contrasting role, bringing certain traits or skills that participants themselves felt they lacked. This created a complementary balance with the participant. Utterances of participants frequently revealed patterns that align with traditional gender role patterns. Mothers, female participants or female partners were often characterized as caring. Fathers, male participants and male partners, on the other hand, were often described as pragmatic and stoic.

### Responses to children’s symptoms

Three main categories of symptom responses were identified: affective, practical and minimizing. These responses all concerned behaviors that were exhibited in reaction to symptoms of children and reflect reinforcement. The same categories emerged in both the descriptions of parents’ responses to participants’ symptoms in childhood and participants’ responses to their own children’s symptoms. Symptom responses by parents were assigned to one of the three categories, based on what was most prominent in participants’ narratives from their childhood. Thereafter, we categorized the symptom responses by participants to their own children, based on what was most prominent in their narratives of the upbringing of their children in their current families. Below, the three categories are discussed in detail.

#### Affective responses

The focus of affective responses (quotes in Table 2) is on alleviating distress through warmth and empathy and fostering the emotional bond. This type of symptom responses involves expressing emotional support and providing comfort to the child (quote P32). Parents in this category listen attentively, validate the child’s feelings and take their complaints seriously (quote P19). There is room to openly communicate about the experienced symptoms and possible causes, whether biological or psychosocial (quote P15). The ill child may be granted certain privileges. For example, a participant described that his mother allowed him to stay home from school and made him special snacks (quote P36). Another participant described that he brings his children a hot-water bottle when they have a stomachache, and takes off from work and coddles them when they are ill (quote P13).

**Table 2:** Quotes related to affective symptom responses.

Parent to participant	Participant to child
P32 I: And can you also remember a time in the past when you were sick, couldn't go to school or something? How that went? P: Yes, that that, then my mother was home and, uh, I know that in high school I had a lot of menstruation complaints. Then I really was sick for a day, and then, yes, then, then, yes, a memory comes to mind suddenly. Yes, I was allowed to stay home and my mother would sometimes clean a bit and, at my aunt's, and then I would come along and lie on the couch, I remember that, or something like that. Yeah, yeah. I: Yes, so then you were allowed to stay home then. And were you also comforted then, or something, or? P: Yes, I was taken care of then. I would get some, well, I would be brought a cup of tea, or something like that, I would be allowed to lie on the couch with a blanket or, uh... P19 P: Oh, I had very, very caring parents. Uhm, yes, I was always taken seriously in that. I was sick regularly of course. My mother in particular was always the one who took care of me. She would get out of bed at night if I wasn't feeling well. I: Yes. P: So no, I, I think in the same way, very loving, yes, well taken care of. P36 I: And when you in the past, because so you used to be sick more often, you said, how did your mother handle that? Or your father when you were younger? P: Oh, very caring. She's [[foreign]], so then you'd get chicken soup and scrambled eggs and everything. So that, no, that was very nice. I: Yeah, yeah. And then you were allowed to stay home from school, or? P: Oh, yeah, yeah.	P32 I: I would also take a bit of time to comfort the child, because yes, that will probably hurt for a moment. And just have a look: how does it look? And maybe it needs a band-aid, and recover a bit. But yes, then also try again, yes, yes. I: Oh yeah, so a little bit, bit like this, only first have a look. P: Also a bit of attention for the emotion, yeah, yeah. P15 P: So, so, really just purely physical assessing, not like: where does it come from? And that, so I do that now. I: Yes. P: Well, why do you have a stomachache? Or well, why is a difficult question, but how come? Or is there something going on? You know, so then... I: Yes, it could be related to something. P: Exactly, so looking a little further than just: oh, your stomach is bothering you. P13 I: Yeah, yeah, hey, and if they have something phy- physical or they're sick or something? What, what, what do you do then? P: Oh. Pam- pamper them. I: Yes. P: Then I pick up a hot water bottle if they have a stomachache or I cuddle up next to them. They always come and cuddle with me. Always. Both of them. Always be there for them, and then I also stop working and then I'm there for them.

*Note.* The participant numbers above the quotes correspond to the numbers shown in the figures.

#### Practical responses

In practical symptom responses (quotes in Table 3), the emphasis is on managing the physical aspects of the illness in a direct, calm and solution-oriented manner (quote P7.1; quote P23). These symptom responses involve the parent addressing the child’s symptoms through concrete actions, such as administering medication, monitoring temperature, giving explanations and normalizing symptoms, or making decisions about seeking medical care (quote P37). The parent tries to assess, based on severity of the symptoms, whether daily activities can be continued or if action needs to be taken. For example, participants described how minor complaints were handled in a straightforward, uncomplicated manner, without placing much emphasis on the symptoms (quote P26). Other symptoms were calmly monitored by parents (quote P7.2).

**Table 3:** Quotes related to practical symptom responses.

Parent to participant	Participant to own child
P7.2 P: And every once in a while it was asked: let's see the finger, because I remember that that that was my middle finger, and yeah, it does get bigger, you should keep an eye on it after all. I: Yes, but that was it actually, wasn't it? Keeping an eye on it, and then, well. P: Very back to earth, very calm! I: Yes. P: Yes, and then action was taken. P26 I: Or if a child has, uh, has a little accident or that… P: Yes, you’ll always have that, of course, but yeah… well… then a band-aid on it and that’s it. I: A band-aid on it and that’s it? [laughs] Yes? P: Yes, something like that, I think, yes. Don’t make it too difficult, no [laughs].	P7.1 P: Hey, if a daughter is sick or if, right, the youngest has, well once has already had corona, then she had 40 degrees fever. I: Yeah. P: Yeah, then we actually stay calm about it. And at one point she was so sick that we still said, well, in all calmness, was still a ba-, yes, she was still a baby actually. Well, we grab her and we put her in the car, bring, we'll go to the ER. Called the ER, get it checked, get it looked at, but she wasn't having trouble breathing or anything. Then I drove there, very calm, I drove there. P23 P: Yeah, yeah, then you see your son lying there on the bed and and injections in him, right, anesthesia. And then I saw him turn white and then I said like: maybe it would be wise to lay him down flat, because I think he's going to pass out [laughs]. Like that. I, I remain quite level-headed in that. P37 I: Yes, have you ever been to the general practitioner with them? P: Yes, I'm somebody, if I don't trust it. I'm, I do go, look, if you have a headache, or you feel good or you don't feel good. Well then you take a paracetamol. If it doesn't go away, well, you stay home for a while, you check the next day and if the next day it still hasn’t passed, then we'll go to the general practitioner.

*Note.* The participant numbers above the quotes correspond to the numbers shown in the figures.

#### Minimizing responses

Minimizing symptom responses (quotes in Table 4) are characterized by a lack of acknowledgment or disregarding of the child’s symptoms. Parents may downplay the severity of the experienced symptoms, encourage the child to carry on with normal activities, or avoid responding to the child’s expressions of discomfort. For instance, the child is told not to whine (quote P13) or exaggerate (quote P22). There is no room for communication about symptom-related distress (quote P13). Parents engage in little to no emotional or behavioral responses in reaction to their child’s symptoms and let children manage on their own (quote P18). The purpose is reducing attention to the symptoms and maintaining routine. No special measures are taken and school and work should be continued as usual (quote P28).

**Table 4:** Quotes related to minimizing symptom responses.

Parent to participant	Participant to own child
P13 P: But I didn’t have to come to my father. I: No. P: He just said, um, “Come on you, no big deal.” I: Yeah. P: I had a broken arm once. I say [whimpering] “My arm doesn't do what it's supposed to do anymore. I'm in pain.” “Eh don't whine, come on, keep going. Then you take it in your other hand.” P28 P: Well, that’s also kind of from how I was raised. Well, that working hard, being sick is basically not an option. I: Okay. P: So if you have a cold or a headache, well then, we just go anyway, we don’t do that. I: To school, to work. P: School, work, yes, there’s basically not really room for that.	P22 I: Yes and can you, did that ever occur? That that that with the kids, that that they were not feeling well or something? Or? P: Yes, yes, yes, a small cut on the finger or something like that. And then she squealed a lot about that. And um, well, “You shouldn't exaggerate, it will pass. Done.” P18 I: How do you assess the severity of your children's illness? P: Um, my wife assesses that, and I always, perhaps often, said, don't complain, carry on, but that, yes, that's in line with... I: But but what do you mean by your wife assesses that, in the sense of? P: Well, she takes it seriously, so if someone, if the boys say, well, I, I, I'm tired or I'm, uh, sick or whatever... She's a bit more understanding. Every time they come in, you look good or you don't look good. How are you feeling? Well, I don't know, while I think, well, it's nice that you're here and... um... um... I: You're still standing, so you must be okay? P: Nice to see you, and the rest is up to you.

*Note.* The participant numbers above the quotes correspond to the numbers shown in the figures.

This type of responses was described the least frequent by participants when addressing their own children’s symptoms. When they did employ minimizing responses, it could come from a clear underlying attitude (i.e., instilling the mentality that discomfort can be overcome through sheer willpower). In other cases, it seemed to be a rather unconscious response or without apparent reason. Minimizing responses varied in tone, ranging from milder forms (i.e., encouraging to maintain routine and not focus on symptoms) to harsher (i.e., implicating that children should not complain when ill).

### Intergenerational transmission

**Fig. 1:**
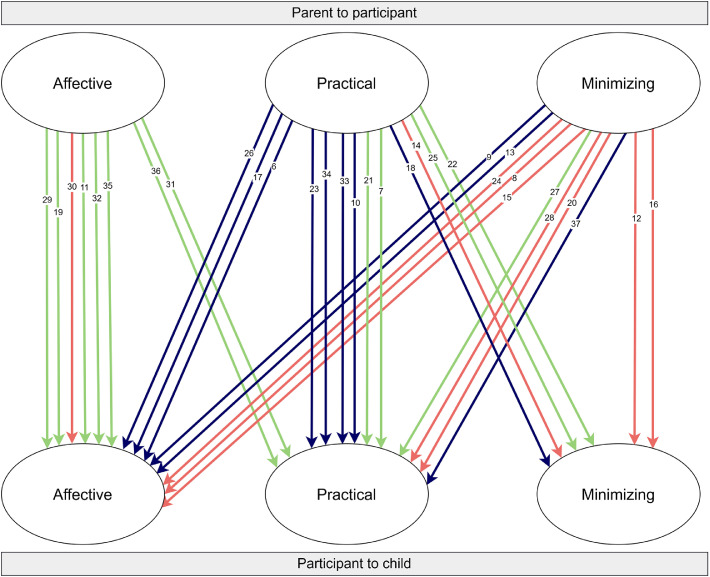
Intergenerational transmission pathways of symptom responses.

*Note.* Colored lines represent evaluations of parental symptom responses: red = negative, green = positive, blue = neutral. Each numbered line represents a participant.

Fig. 1 visualizes the pathways of intergenerational transmission of symptom responses. To aid interpretation of Figure 1, the upper circles represent categories of parental symptom responses towards participants, and the lower circles represent participants’ responses towards their own children. Each arrow traces an intergenerational pathway between these categories (affective, practical, minimizing), with one arrow per participant. The coloring reflects whether parental responses were evaluated as negative, positive, or neutral.

A number of patterns emerged from the data. Both adopting similar and divergent symptom responses compared to parents occurred. We categorized evaluations of parental symptom responses by participants as positive, neutral or negative. Neutral was assigned when participants were neither markedly positive nor negative. Categorizations were made based on the ways participants spoke about their parents’ behavior and explicit answers provided to questions by the interviewers about participants’ opinions of their parents’ responses. All three categories of evaluations occurred. Evaluations of parental responses did not always clearly relate to whether participants adopted similar or divergent responses towards their own children’s symptoms.

Participants whose parents employed affective symptom responses mainly shared positive evaluations of this. None of these participants adopted minimizing symptom responses to their own children’s symptoms. Instead, they either responded in a practical way or, similar to their parents, in an affective way. Some explicitly explained that they mirrored their parents’ approach because they held positive memories of how their symptoms were responded to during childhood:

*P35*

*P: So when we were sick, then yeah, then then it was always very… Pleasant. Well, [makes air quotes] as pleasant as being sick can be, but then… It was actually quite nice, the way she approached it.*
*I: Yeah, because how did she approach it?*
*P: Well, just like I said. Very caring, a hug, taking care of you, always with a cup of tea or something to drink or eat. An apple, everything prepared, so that was, eh, yeah, she still does that.*
*I: And how do you do that now or? Well, probably not anymore now that the children have moved out. But when when your children were little, how did you approach it when they were sick?*
*P: Well, the same.*
*I: Yeah.*
*P: Precisely because I found that so nice, I think. You want to do that yourself too. So then, then we’d give, well, nice and cozy on the couch. Cozy blanket. An apple, you know, like that. That was, that’s what I had too.*

Parental practical responses were generally evaluated as neutral or positive. There is variation in the type of responses that participants with parents in this category employed in managing their own children’s symptoms, distributed across all three categories. One participant explained the theory behind his parents’ practical responses and told he applied the same sort of responses:

P7

*P: “Everything okay? Yeah, now get back on the bike!” Then off you go again.*
*I: Yeah.*
*P: So yeah, no, that was also very—*
*I: Yeah, and what do you think about that?*
*P: No, I’m like that myself too. I think that as soon as I give more attention, so how’s it going, then I’m actually conditioning my child to a… Well. If he cries and I say “Oh, how are you?” and I give him a lot of attention. Yeah, if you give something attention, you’re actually rewarding it, and then that child will cry more.*
*I: Yeah.*
*P: If the child isn’t crying yet and nothing’s wrong, then it’s: “Hey, come on, get up. You’re doing great.” and positively reward, especially for the behavior.*
*I: Yeah, so you’re very consciously doing that, based on the belief that that’s how it works.*
*P: Yeah, I really got that from my parents. Yes.*

Participants whose parents employed minimizing symptom responses nearly all expressed negative or neutral evaluations of this. Almost all of these participants employed different types of responses to their own children’s symptoms, both affective and practical. Some participants expressed that adopting different responses than their parents was linked to the negative experiences they had with their parents’ responses:

P28

*I: I have a, a, a short little story. Imagine a child learning to ride a bike, the child falls and starts crying, and the parent standing nearby says: “Falling is part of it, come on, get back on and keep going.” What do you think of that reaction?*
*P: I think that’s really bad.*
*I: Yeah? Why?*
*P: Yeah, because, well, that actually sums up exactly how I was raised and how I don’t want it to be for [daughter’s name], so to speak.*
*I: Hmm.*
*P: Right, because I think then you’re not paying attention to the fact that someone can really be in pain, and because for her it might be, she might be really shocked or, right, hurt herself, and, and, and well yeah, this kind of sums it all up, so to speak.*

### Setting an example

Participants were asked about examples they aimed to set for their children with regard to approaching illness, reflecting modeling. Most participants shared their will to set the right example and reflected upon this. The examples they described are not primarily behavioral, but rather reflect the participants’ underlying values about what they consider important to model to their children regarding approaching illness. We identified three main categories of value-driven examples that participants set for their children in approaching illness: transparent and vulnerable, stable and calm, and tough and perseverant. Like the symptom responses, the examples of participants were assigned to one of the three categories, based on what was most prominent in their narratives of the illness-related upbringing of their children. Below, we described the three categories in detail.

#### Transparent and vulnerable

This category reflects participants’ intention to model a transparent approach to illness to their children. Participants described the importance to communicate openly about their own illness experiences and emotional distress associated with those illness experiences. By doing so, they aimed to show their children that it is acceptable to express vulnerability and seek help and support when feeling unwell. Participants also emphasized the need to take symptoms seriously and listen to one’s body.

P15

*I: Is that something you also find important to pass on to your children, like…?*
*P: Yes. Well, at the very least: seek help.*
*I: Yeah.*
*P: You don’t have to solve everything on your own, because sometimes you just can’t, you get stuck in a loop. I recognize that from my own experience, like, well, not talking to anyone about it because you have to pretend everything’s fine. No, seek help!*

#### Stable and calm

The category stable and calm refers to a model of a composed, balanced approach to illness. Participants described how they strived to remain emotionally steady and maintain a sense of calmness when experiencing symptoms. By doing so, they aimed to create a predictable and reliable environment in which illness is acknowledged but not dramatized. Participants wanted to show their children that symptoms are a natural part of life that can be managed with appropriate measures and without panic.

P7

*I: So that that that, yeah, the the good example, right, that I asked about, yeah, that–*
*P: Yeah, but that you do stay calm and my oldest was of course just there. She also saw that her little sister was sick.*
*I: Yeah, and then she also sees how you deal with that.*
*P: Yeah, and well, I say well, then I’ll take her with me, then I quietly put her in the car and well, give give a quick kiss, well and then I calmly drove that way [to the hospital].*
*(…)*
*P: Yeah, you do try to pass on that calmness.*

#### Tough and perseverant

This category refers to participant’s intention to model strength and perseverance when facing illness themselves. Participants in this category described demonstrating a “tough” attitude, often minimizing their discomfort and continuing daily activities despite feeling unwell. Participants strived to show their children that experiencing symptoms does not require withdrawal or vulnerability, but rather, that it is possible to endure discomfort.

P16

*P: What eh, what’s a good example? Eh, in our family it’s like, don’t complain too quickly, right, that eh, you just have to keep going. There are worse things. Maybe we’re limping a bit, but the world, eh, doesn’t turn upside down because of that. No drama.*
*I: Yeah. And how, and how do you pass that on as an example to your children? Or how, eh?*
*P: Well yeah, look, my kids have known me for a while now, so, they don’t come to me with that kind of nonsense.*

Participants expressed varying degrees of certainty about the example they set for their children. Some were confident they gave the right example, which they believed would yield positive effects. Others, on the other hand, expressed uncertainty about what the right example entails, their ability to set it and potential pitfalls in their modeling.

**Fig. 2:**
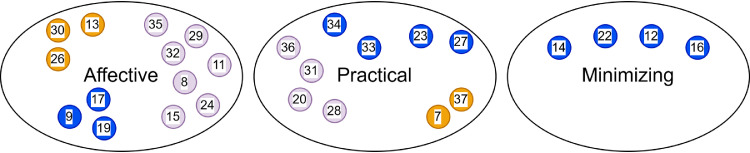
Value-driven examples in relation to symptom responses.

*Note.* Each numbered circle represents a participant. Purple = transparent and vulnerable, yellow = stable and calm, blue = tough and perseverant. Not all participants elaborated on their value-driven examples and could therefore not be included in this figure.

Figure 2 shows the relations between value-driven examples and symptom responses. Each numbered circle represents a participant, and the colors correspond to the three types of value-driven examples. The placement of circles within each response category visually depicts how aspired examples link to participants’ symptom response type. The thematic analysis suggested potential matches between the three categories of symptom responses and the value-driven examples, with affective responses aligning with a transparent and vulnerable example, practical responses with a stable and calm example, and minimizing responses with a tough and perseverant example. However, as Fig. 2 shows, this seems to be only entirely the case for participants with minimizing symptom responses in our sample, who all aim to set the example of being tough and perseverant. Participants who employ affective and practical symptom responses described value-driven examples of all three categories.

### Deviant case analysis

A few cases seem to deviate from the general emerged patterns. First, deviant in Fig. 1, one participant (P27) evaluated their parent’s minimizing symptom response as positive, while all other participants evaluated minimizing responses as negative or neutral. This participant explained that he preferred to be left alone and not receive attention when he was experiencing symptoms:

P27

*P: Yeah, I, when I, when I was sick, when I was really sick and then I didn’t feel like being around people, and just go away, dark, quiet.*
*(…)*
*P: Everyone and everything, just go away!*

Second, deviant in Fig. 1, two participants (P12 and P16) expressed negative evaluations of their parents’ minimizing symptom responses, yet employed minimizing symptom responses to their own children’s symptoms too. This contrasts with the broader trend of employing different symptom responses to own children, often in reaction to negative experiences with parental minimizing responses. Both of these participants shared that they missed warmth in their childhood. Their parents were unable to respond to their symptoms appropriately, which was at least partly attributed to the parents’ mental health problems. One participant explained that the lack of parental warmth had made himself tougher too:

P16

*I: And uh, yeah, even though your own mother was ill for a long time during your childhood, or maybe still, uh. Yes, by the way, do you think, could that have had an impact?*
*P: Yes! Well, the illness, right. It made you really tough. Tougher. Because you missed a bit of warmth growing up. And here, what we do here, it’s a, a kind of act really, at the office, and that’s, yeah, that’s just something you’ve learned.*
*I: How do you mean? Because of what you missed when your mother was ill?*
*P: It’s stronger, you are, yeah, it made you more businesslike. Also within the family.*

Third, deviant in Fig. 1, one participant (P30) evaluated their parents’ affective responses as negative, yet employed affective responses to their own children too. This deviates from the other participants whose parents employed affective symptom responses. This participant described their parents as overly protective, displaying disproportionate emotional reactions when the participants had symptoms. Their parents’ excessive concern often led to the participant being discouraged from engaging in regular daily activities or sharing their symptoms:

P30

*P: My father was also the type… Well, I was somewhat held back, let me put it that way. Like, “Well, go on, you're not feeling well? Do you have a headache? Just lie down, take some paracetamol, and lie down.” Okay, he was very concerned, overprotective, really. I’m the same way with my daughter, but only to a certain extent. I think I’ve learned from those experiences. But yes, I was in that way, like, yes—*
*I: Do you mean—?*
*P: I didn’t feel well, “Oh, just stay home from school.” And looking back, I sometimes think, maybe I should have gone to school anyway.*

Fourth, deviant in Fig. 2, three participants (P9, P17 and P19) aimed to set a tough and perseverant example for their children, but employed affective symptom responses. This seems somewhat contradictory. For these participants, there appears to be a discrepancy between the toughness they intended to model and the more caring behaviors they displayed in reaction to their children’s illness. One participant related his affective responses to a prior serious illness experience of his child, which made him particularly protective. Another participant mentioned that her nurturing approach stemmed from the fact that her young child was not yet able to communicate verbally, making it more difficult for her to assess the severity of symptoms:

P19

*P: Uh. Yes, I try to set a good example, but yeah, what is a good example, right? [laughs] So I’m someone who’s pretty quick to say: just push through and it might get better. I think that’s good.*
*(…)*
*I: And what, what do you do when your son is sick?*
*P: Well, I find that more difficult. Because I think, with him I can’t really assess that of course, right, how he’s truly feeling. So I keep, keep him home more quickly. Like, oh, he goes to daycare three days a week, and then I tend to keep him home sooner. Then I think: ah, I feel bad if he’s feeling really, really unwell and has to go to daycare.*
*I: Hmhm.*
*P: And he can’t tell us yet, right?*

## Discussion

This qualitative study explored how illness approaches are shaped in parent-child interactions and transmitted across generations. We identified three distinct symptom response types, reflecting reinforcement, in two generations: affective, practical and minimizing. Both mirroring parents’ responses and diverging occurred in participants’ approach to their own children’s illness. Affective and practical responses were mostly evaluated as positive or neutral. Minimizing responses were generally evaluated as negative or neutral and linked to deliberate efforts to adopt different responses to own children’s symptoms. Participants articulated value-driven examples they aimed to model for their children, categorized as transparent and vulnerable, stable and calm, or tough and perseverant. These did not always align with the way they responded to their children’s symptoms, revealing contradictions between reinforcement and intended modeling.

Although we did not incorporate the Adult Responses to Children’s Symptoms (ARCS) questionnaire in the development of the interview guide or coding scheme beforehand, the three symptom response categories identified in our study broadly correspond to the subscales found in the ARCS: Protectiveness, Encouragement/monitoring, and Minimization (Claar et al., 2010; Van Slyke & Walker, 2006). The ARCS assesses parental responses to children with chronic pain conditions, whereas our study explored responses to a broad spectrum of both short-lived and persistent somatic symptoms in the general population. The correspondence between the two suggests that parental responses may generalize across populations and symptom types.

Affective responses relate to Protectiveness, including behaviors such as relieving the child from responsibilities and granting special privileges. Most participants in our sample evaluated affective responses positively, characterizing their parents as warm and supportive, for example by offering small comforts like chicken soup and a blanket on a sick day. However, ARCS literature associates protectiveness with elevated child impairment and longer symptom duration (Claar et al., 2008; Simons et al., 2008), presumably because these behaviors signal the child that their symptoms are concerning. Notably, two participants in our study described their parents’ responses as *over*protective. A possible explanation is that the ARCS does not differentiate between sensitive emotional support and prolonged disproportionate protectiveness, while these may have distinct effects on children. Parental overprotection has been shown to predict future physical symptoms, whereas parental warmth and affection predict lower health risks (Carroll et al., 2013; Janssens et al., 2009).

The practical responses identified in our study resemble the ARCS encouragement/monitoring subscale, which encompasses providing distraction, encouraging to engage in activities and checking on the child. Experimental research, including studies using the cold pressor task, suggests such responses may be most beneficial in reducing symptom burden, especially if they shift the child’s attention away from the symptoms (Walker et al., 2006). Moreover, monitoring and assessing illness severity is essential for determining appropriate care for the ill child. Whereas underestimation and overestimation of symptom severity can lead to missed treatment opportunities or unnecessary restrictions and interventions, accurate parental judgement can improve child outcomes (Langer et al., 2009; Yoos et al., 2003). Our participants evaluated practical responses most often as neutral. This may reflect the functional nature of these responses, focused on normalization rather than emotional comfort. Importantly, most participants in our study described experiences of occasional illness rather than chronic conditions. In such contexts, a warm and comforting response may be perceived as more appropriate than encouraging activity engagement. This distinction is important when comparing our results to the ARCS literature which is focused on chronic pain.

Minimizing responses correspond to the ARCS minimization subscale, entailing criticism or dismissal of symptoms and encouraging continuation of routine as usual. Minimization has been associated with increased physical symptoms (Claar et al., 2008). When parents minimize, children might (unconsciously) amplify their symptoms in an effort to be validated. Alternatively, children may feel undermined or neglected, which could increase symptom severity through somatization (Claar et al., 2008). In line with this, it has been shown that parental underestimation of symptoms is associated with increased symptom prevalence years later (Hogendoorn et al., 2023). These findings match the negative evaluations participants frequently provided for these responses. Our interviews also revealed a more nuanced picture then the rather rigid ARCS categorization seems to suggest. Some participants described milder forms of minimization, related to encouraging perseverance or maintaining routine, which could be viewed as promoting resilience. Our qualitative data offer detailed insights beyond the ARCS literature, suggesting that while minimizing may often be considered as invalidating, it is not inherently negative in all contexts.

Importantly, prior research showed that children’s emotional functioning moderates the relation between both protectiveness and minimization and symptom-related outcomes. This implies that the influence of parental responses also depends on individual child factors (Claar et al., 2008), which supports our finding that similar parental responses can be evaluated in various ways. Our findings also show variability in whether and how participants mirrored or diverged from their parents’ approaches. Some made deliberate choices to either emulate or diverge based on their evaluations of their parents’ approaches, while others described more intuitive decisions. Additionally, many participants highlighted the role of partners in shaping responses to their children’s symptoms. These findings underscore the complexity of intergenerational transmission of illness approaches. They suggest that such transmission is not merely imitative, but also involves reflective processes and is shaped by personal traits, memories, and contextual factors. As such, similar parental symptom responses are not evaluated in the same way by all individuals, and positive or negative evaluations do not necessary lead to mirroring of or diverging from this response type towards one’s own children. Partner dynamics, the quality of family relationships and illness experiences in the family appear to play a role in forming and transmitting an illness approach. This study complements existing quantitative research on intergenerational transmission of illness-related behavior based on questionnaires or observations. It provides an in-depth layer to the understanding of such transmission, by revealing how individuals subjectively reflect on and engage with illness approaches in the family.

The value-driven examples participants shared somewhat relate to the concept of “good-parent beliefs”, which refers to parents’ personal sense of what being a good parent to an ill child implies. Developed in the context of serious illness (Feudtner et al., 2015), these beliefs influence parents’ medical decision making and how they aim to behave around their ill children. Examples of good-parent attributes are making sure the child feels loved and focusing on the child’s health. Our findings extend this literature by showing how values also shape everyday illness-related modeling in non-clinical populations, and are thus not only of influence in serious medical contexts but also in subtle interactions around minor illness.

While both parental modeling and reinforcement of illness behavior have been extensively studied and proven to influence child symptom experiences (Goodman & McGrath, 2003; Hogendoorn et al., 2024; Janssens et al., 2009; Simons et al., 2008; Sobol-Kwapińska et al., 2020), the extent to which these social learning mechanisms align has received less attention. Our results suggest that they do not always coincide. Although the symptom responses and examples appear to correspond conceptually, consistent alignment was only observed among participants with minimizing responses. How this degree of alignment affects the shaping of children’s illness approaches remains unknown and warrants further investigation. Similar discrepancies have been observed in other health-related domains. For example, research on parental smoking found that smoking status was related to setting smoking rules, but did not influence the frequency and type of anti-smoking messages they delivered to their children. Parental smoking and setting rules were linked to adolescent smoking, while anti-smoking messages and monitoring were not (Kodl & Mermelstein, 2004). Another study on the intergenerational transmission of risky behaviors reported that parental reinforcement was the strongest predictor of children’s current risky behavior, whereas parental modeling had a stronger influence on children’s anticipated behavior in adulthood (Morrongiello et al., 2008). These findings highlight the complex relation between modeling and reinforcement, and suggest that their effects may not only differ in nature but also across time and developmental stages. Together, these insights underscore the importance of considering both mechanisms when examining how health- and illness-related approaches are intergenerationally transmitted.

### Strengths and limitations

A key strength of this study, enhancing credibility, is the mixed-method sampling approach, which integrated quantitative epidemiological data into purposive sampling. This strategy enabled the inclusion of participants with varied ages, occupations and familial illness histories. Additionally, a deviant case analysis was performed to reduce the risk of confirmation bias (Hennink et al., 2020) and member checking allowed participants to validate transcripts of the interviews. Transferability of research results was supported by the diversity of our sample. Unlike many qualitative health-related studies that tend to overrepresent young, highly educated women (Polit & Beck, 2008), our sample was more balanced. Just over half of the participants was male and a similar proportion had a low to medium level of education. Consequently, we could capture varied experiences and perspectives, contributing to a nuanced understanding of patterns and exceptions. Another strength lies in the multidisciplinary composition of the research team, consisting of six researchers with diverse experiences and areas of expertise, which strengthened dependability (Shenton, 2004). We engaged in regular collective team discussions, which enabled us to address differing interpretations, personal reactions, and disciplinary vocabularies, to maintain a consensus throughout the study. We ensured that our generation of themes was driven by a shared understanding of the participants’ narratives, instead of one dominating disciplinary lens. Trustworthiness was further established by keeping an audit trail throughout the research process (Shenton, 2004).

Some limitations should also be acknowledged. First, selection bias may be present, with only those participating that had intrinsic interest in the study topic. However, our analyses revealed substantial variation in how central the topic of illness was to participants’ lives. Given their long-term involvement in the Lifelines cohort, some subjects may have participated in this add-on study out of a sense of duty rather than personal relevance. Second, assigning parental and participant symptom responses to one of the three categories is a simplification of reality. In practice, individuals may exhibit aspects of multiple response styles, which could not be fully captured by our categorization. Each parent could also show a different response type, in which case we chose the category that was most prominent in the narratives. Third, although our sample is varied in socioeconomic status, geographical location, sex and age, it is important to note that Lifelines mainly comprises participants of Dutch origin (Klijs et al., 2015). The cohort is roughly representative of the adult population in the northern Netherlands (Klijs et al., 2015), but populations in other regions of the country are more culturally diverse. As such, experiences and perspectives of individuals from different cultural backgrounds could differ, and our findings may not fully capture the variation of lived experiences of these groups. Moreover, cultural norms around symptom interpretation, communication, and health-related decision-making may influence how illness approaches are formed and transmitted between generations. Our findings should therefore be interpreted in the Dutch sociocultural context, in which norms as autonomy are valued, and in which general practitioners serve as gatekeepers of the healthcare system. This may differ from other countries or cultural settings. Further research is needed to explore how intergenerational patterns of illness approach manifest in more culturally diverse contexts. Lastly, it should be noted that participants’ evaluations of parental approaches are retrospective. As shown in previous research, pain memories are reconstructed and influenced by social contexts (Noel et al., 2015). Participants’ narratives about their childhood may be shaped by later interactions with their parents, current beliefs or their own caregiving experiences. Narratives of participants’ approaches towards own children reflect more recent memories, but could be affected by social desirability. We attempted to limit this by establishing rapport, emphasizing there were no wrong answers, and giving the participant the opportunity to stop the interview at any time without providing a reason for this.

### Implications and future directions

Few studies have qualitatively explored illness-related parent-child interactions in non-clinical samples. Our findings shows that intergenerational transmission of illness approaches is a complex and often reflective process, shaped by individual experiences and varying widely across families. Because participants’ accounts of their parents’ responses were retrospective, any implications for long-term child outcomes resulting from this study should be interpreted with caution. Further research is needed to establish how illness approaches evolve over time, ideally through longitudinal designs that follow families for longer periods. Such studies could also investigate whether certain illness approaches predict more or less favorable symptom-coping in children, offering insight into protective and risk factors. This would be valuable given the societal costs of non-adaptive coping, related to school and work absenteeism and increased healthcare utilization (Joustra et al., 2015; Konnopka et al., 2013; Logan et al., 2008). Additionally, conducting interviews with multiple family members, for instance, both parents and children or siblings, could further illuminate how illness-related values are shared and shaped within families. The role of partner influence or co-parenting dynamics may also be explored in this respect. Moreover, since our study focused on intergenerational processes and parenting, we did not include how participants approach own illness. This aspect may play a significant role in modeling illness-related behavior and should be considered in future research. Deeper reflections on how participants’ childhood experiences shaped their current intentions in illness-related modeling, including exploration of specific contexts in which these experiences were evaluated as helpful or harmful, could also be explored in this respect. Furthermore, future quantitative studies could aim to distinguish between sensitive emotional support and overprotective parental responses, as these may have different implications for child well-being. Finally, multi-method designs that integrate quantitative and qualitative approaches could offer a more comprehensive understanding of how illness approaches are transmitted and interpreted within families. For example, combining interviews with validated instruments like the ARCS may help clarify how subjective experiences relate to more objective behavioral outcomes and could provide information on long-term influences of parental responses.

This study’s results may aid in improving family-based interventions for individuals presenting with physical symptoms. Understanding how parents respond to their child’s symptoms could help healthcare professionals tailor guidance, for instance upon child discharge from hospital. Importantly, clinicians should be attuned to the balance between emotional sensitivity and overprotection. Helping parents reflect on this balance and adapt their responses to meet their child’s needs may foster children’s well-being. Family therapy could also benefit from exploring how parents’ own childhood illness-related experiences translate into their caregiving approaches, potentially breaking adverse intergenerational cycles. Lastly, the findings suggest that modeling and reinforcement are distinct yet interrelated mechanisms in social learning of illness approaches. Clinicians should consider parents’ values as well as their behavioral responses in treatment of childhood somatic symptoms. Making inconsistencies in these values and actual responses explicit may encourage more cohesive caregiving practices. Psychoeducation in primary care and public health settings could focus on aligning parental values with their day-to-day responses. This would provide parents with practical strategies to model the behaviors they wish to promote, and reinforce adaptive coping in their children.

## Data sharing

Lifelines data will not be shared publicly. Access to the Lifelines data is organized according to a strict data access procedure. For all types of access, a research proposal must be submitted for evaluation by the Lifelines Research Office. The evaluation is performed to align the goals of the researchers with the goals of Lifelines (which are in turn aligned with the informed consent form signed by Lifelines participants). Further information on Lifelines data can be obtained by contacting the Lifelines Research Office (https://www.lifelines.nl).

## Declaration of competing interest

The authors declare that they have no known competing financial interests or personal relationships that could have appeared to influence the work reported in this paper.

## Appendix Supplementary materials

Image, application 1

Image, application 2
