# Supplementary material for: Roots of responses: A qualitative study on intergenerational transmission of illness approaches
Source: Int J Clin Health Psychol. 2026 May 30;26(2):100692. doi: 10.1016/j.ijchp.2026.100692 (PMC13241775; doi:10.1016/j.ijchp.2026.100692)
Supplement: Supplementary file 2 [file mmc2.docx]

11-05-2026

Dear Prof. Wu,

Thank you for giving us the opportunity to submit a revised version of our manuscript entitled “Roots of Responses: a qualitative study on intergenerational transmission of illness approaches” (IJCHP-D-25-00621) and for sending us the reviewer’s new suggestions.

We have revised the manuscript according to the reviewers’ comments. Below, we answer the issues raised. Specifically, following Reviewer #1’s comment, we have now excluded the five pilot interviews from the analysis.

We thank the reviewers for their time and hope that you will find the revised manuscript suitable for publication in the International Journal of Clinical and Health Psychology. All authors have seen and approved the final manuscript text.

Sincerely, on behalf of the authors,

Elske Hogendoorn

Author response

Reviewer #1

**The revised manuscript has addressed most of the queries and comments raised in the previous review. However, it still fails to resolve the major methodological flaw previously identified—the inclusion of the five pilot interviews in the analysis. In their response, the authors argue that "the modifications to the interview guide after the pilots were minor" and that their inclusion did not affect the analytic outcomes. These arguments, however, sidestep the core issue: by incorporating the pilot interviews, the authors compromise—and potentially contaminate—the original purposive sampling design established through the Lifelines Cohort Study.

Moreover, I find no compelling justification for maintaining these pilot interviews, especially given the authors' own assertion that their inclusion did not alter the analysis. The authors further contend that integrating pilot data is "consistent with standard practice in qualitative research." While this may hold true for studies lacking a defined sampling strategy, such as snowball or referral sampling, it is inappropriate in this case, where a clear purposive sampling design has been delineated. The five pilot interviews simply do not conform to that framework.

I therefore uphold my earlier position that the pilot interviews should be excluded from the analysis and discussion. I fundamentally disagree with the authors' methodological approach and will undertake a full re-evaluation of the manuscript only once this substantial issue has been adequately addressed.**

We thank the reviewer for their time and willingness to review the paper. We have addressed the concern regarding the pilot data. The pilot interviews have now been excluded from all analyses.

Reviewer #2

**The authors have responded to all my comments, and I have no further comments.**

We thank the reviewer for their time and for confirming that all comments have been satisfactorily addressed.
